# A novel method for downstream characterization of breast cancer circulating tumor cells following CellSearch isolation

**DOI:** 10.1186/s12967-015-0493-1

**Published:** 2015-04-21

**Authors:** Henrik Frithiof, Charlotte Welinder, Anna-Maria Larsson, Lisa Rydén, Kristina Aaltonen

**Affiliations:** Division of Oncology and Pathology, Department of Clinical Sciences Lund, Lund University, Lund, Sweden; Skåne Department of Oncology, Skåne University Hospital, Lund, Sweden; Division of Surgery, Department of Clinical Sciences, Lund University, Lund, Sweden; Department of Surgery, Skåne University Hospital, Lund, Sweden

**Keywords:** Circulating tumor cells (CTC), Metastatic breast cancer, CellSearch, Biomarkers, Estrogen receptor (ER), Human epidermal growth factor 2 (HER2), Immunofluorescence

## Abstract

**Background:**

Enumeration of circulating tumor cells (CTCs) obtained from minimally invasive blood samples has been well established as a valuable monitoring tool in metastatic and early breast cancer, as well as in several other cancer types. The gold standard technology for detecting CTCs in blood against a backdrop of millions of leukocytes is the FDA-approved CellSearch system (Janssen Diagnostics), which relies on EpCAM-based immunomagnetic separation. Secondary characterization of these cells could enable treatment selection based on specific targets in these cells, as well as providing a real time window into the metastatic process and offering unique insights into tumor heterogeneity. The objective of this study was to develop a method for downstream characterization of CTCs following isolation with the CellSearch system.

**Methods:**

An *in vitro* CTC model system focusing on clinically useful treatment predictive biomarkers in breast cancer, specifically the estrogen receptor α (ERα) and the human epidermal growth factor receptor 2 (HER2), was established using healthy donor blood spiked with breast cancer cell lines MCF7 (ERα^+^/HER2^−^) and SKBr3 (ERα^−^/HER2^+^). Following CTC isolation by CellSearch, the captured CTCs were further enriched and fixed on a microscope slide using the in-house-developed CTC-DropMount technique.

**Results:**

The recovery rate of CTCs after CellSearch Profile analysis and CTC-DropMount was 87%. A selective and consistent triple-immunostaining protocol was optimized. Cells positive for DAPI, cytokeratin (CK) 8, 18 and 19, but negative for the leukocyte-specific marker CD45, were classified as CTCs and subsequently analyzed for ERα and HER2 expression. The method was verified in breast cancer patient samples, thus demonstrating its clinical relevance.

**Conclusions:**

Our results show that it is possible to ascertain the status of important predictive biomarkers expressed in breast cancer CTCs using the newly developed CTC-DropMount technique. Downstream characterization of multiple biomarkers using a standard fluorescence microscope demonstrates that important clinical and biological information may be obtained from a single patient blood sample following either CellSearch epithelial or profile analyses.

**Trial registration:**

Clinical Trials NCT01322893

## Background

During the last decade, enumeration of circulating tumor cells (CTCs) in peripheral blood was established as a prognostic tool for predicting time-to-recurrence and survival in metastatic and early breast cancer, as well as in several other cancer types [1-4]. The gold standard technology, and the only platform implemented on a larger scale, is the FDA-approved semi-automated CellSearch technology (Jansen Diagnostics, Raritan, NJ, USA). In CellSearch, enriched EpCAM-positive CTCs are defined as nucleated cells positive for cytokeratin (CK) 8, 18 and 19, but negative for the leukocyte-specific surface protein, CD45. However, the field of CTC research is now moving beyond solely quantifying cells in peripheral blood. Phenotypic and molecular characterization of CTCs has the potential to provide clinically important information from an easily accessible blood sample, a ‘liquid biopsy’. Serial blood sampling followed by molecular characterization can provide insights into tumor progression and enable early detection of treatment resistance.

In breast cancer, assessment of estrogen receptor α (ERα) status in the primary tumor is crucial in classification and treatment prediction [5]. Determining receptor status identifies patients eligible for endocrine therapy, which remains the mainstay adjuvant treatment for ERα^+^ breast cancers, either as monotherapy or in conjunction with chemotherapy. Although an ERα^+^ primary tumor is a common trait and found in approximately 80% of patients with primary breast cancer, it is no guarantee for a favorable outcome following endocrine treatment as recurrence rates of 19–41% are observed at 10 years following 5 years of tamoxifen [6-8]. Moreover, in metastatic breast cancer, approximately 40–50% of patients fail to respond to endocrine treatment, despite an initially positive assay [9]. The causes of this considerable inconsistency are multifactorial and have not been entirely elucidated, but discordance in ERα status between the primary tumor and involved lymph nodes or distant metastases has been established in 6–30% of studied cases and may contribute to treatment resistance [10-14]. In fact, this phenotypical shift is associated with significantly shorter median survival for patients with metastatic disease when compared with consistent ERα-positivity in disease progression [10]. Additionally, it has been reported that the majority of CTCs in patients with ERα^+^ primary tumors are in fact ERα^−^ prior to therapy, with a concordance of less than 30% [15-17].

Human epidermal growth factor receptor 2 (HER2) is a tyrosine kinase receptor encoded by a proto-oncogene located on chromosome 17 (17q12), and is the second most important predictive biomarker in breast cancer [18,19]. Amplification of this gene occurs in approximately 10–30% of primary breast cancers, correlating with poor prognosis and an aggressive phenotype [18,19]. This subgroup of patients benefits from immunotherapy with an HER2-targeted monoclonal antibody, trastuzumab, in combination with chemotherapy in adjuvant, neoadjuvant and metastatic settings [20,21]. Similar to the dynamic progression observed in ERα^+^ tumors, the HER2 status of metastases can differ from that of the primary tumor [22]. Discordance has been observed in 7–14% of studied cases [11,23-25]. It has been shown that patients with HER2^−^ tumors might acquire HER2 amplification during disease progression, as demonstrated by isolation of HER2^+^ CTCs in patients with an HER2^−^ primary tumor [26-29]. Another explanation for the discrepancy in biomarker expression between primary tumors and CTCs may be tumor heterogeneity. Tumor clones shed into the blood stream are more likely to represent those with most malignancy, exemplified by HER2-amplified clones, despite the primary tumor being diagnosed as HER2 normal. These patients are less likely to receive HER2-targeted treatment, although a complete or partial response has been observed in selected cases [30]. Two prospective trials including patients with HER2-negative primary tumors and HER2-positive CTCs are currently open for recruitment and aim to elucidate whether trastuzumab will have a beneficial effect on these cases [31].

Thus, treatment decisions based on the phenotype of the primary tumor alone might omit critical facts relevant to the prognosis and choice of treatment. Biopsies from metastatic sites are not always available for practical reasons and are inevitably accompanied by an invasive procedure. CTCs are easily accessible from a normal blood sample, and since CTCs are shed from multiple metastatic sites as well as from the primary tumor, characterization of these cells could provide important information for treatment prediction.

The aim of this study was to establish a method for downstream characterization of multiple treatment predictive markers expressed by CTCs after CellSearch-based selection, without the necessity of additional patient samples. Validation of the method in samples from patients with metastatic breast cancer highlights the potential of the clinical utility of this technique.

## Methods

### *In vitro* model

Breast cancer cell lines MCF7 and SKBr3 were obtained from the American Type Culture Collection (ATCC/LGC Standards GmbH, Wesel, Germany) and were used to establish an *in vitro* model system for CTC characterization following CellSearch isolation. MCF7 expresses ERα but is negative for HER2 amplification. Contrary, SKBr3 cells are HER2-positive and negative for ERα. MCF7 cells were grown in a 5.0% CO_2_ incubator under UV-light at 37°C in culture vessels containing 5 mL MEM/EBSS (HyClone Laboratories, Inc., Utah, United States) medium supplemented with 1% sodium pyruvate, 1% non-essential amino acids, 10% fetal bovine serum (FBS) and 1% penicillin streptomycin mixture (Pen-Strep) for MCF7, and RPMI 1640 (HyClone Laboratories, Inc.), while SKBr3 cells were cultured under the same conditions in 5 mL MEM/EBSS plus 10% FBS and 1% Pen-Strep. Harvesting of cells was performed at approximately 80–90% confluency after 5–10 min trypsinization.

Healthy donor blood samples were processed within 24 h from withdrawal, and spiking of cells occurred in conjunction with subsequent CellSearch analyses. Two different spiking methods were used. First, dilution of cells resulted in approximately 2000 cells per 7.5 ml blood, and using the CTC-DropMount technique (described below), approximately 200 cells were applied to 10 individual slides, which were later used in the optimization of staining procedures. Second, to ascertain the recovery rate of the method, a specific number of cells were harvested individually with a 10 μL pipette under a bright-field microscope equipped with a standard achromatic × 10/0.25 objective. In detail, a fraction of the cell culture was transferred to a Petri dish containing cell culture medium. While observing the cell culture suspension through the eyepieces of the microscope, suitable individual cells were selected and carefully extracted using a 10 μL pipette before transfer to a healthy donor blood sample. Since the process is continuously monitored by microscopy, one can confirm that the cell has been properly extracted. Reference values of 5, 15, and 50 cells were selected. Independently collected duplicates of each of the three respective cell quantities were added to 7.5 mL of healthy donor blood samples and processed according to the specified method. The agreement between the measured results and the reference values was calculated to define the recovery rate.

### Fixation of samples using CTC-DropMount

CellSearch Profile (Jansen Diagnostics) analysis was performed according to the manufacturer’s protocol, which involves enrichment of CTCs with magnetic ferrofluid-associated anti-EpCAM antibodies but no consecutive staining. The enriched samples were mounted on slides using a specific procedure developed in-house, CTC-DropMount. The solution containing isolated CTCs (approximately 900 μL) was transferred to an 1.5 mL Eppendorf tube and placed in a magnetic tray. After 10 min incubation, the non-adherent solvent was extracted. The cells were resuspended in 10 μL 1 × PBS, mounted on superfrost slides (ThermoScientific, Germany) and incubated at 37°C for 30 min. Fixation was accomplished by immersing slides in pure methanol for 5 min. The samples were stored at −20°C.

**Figure 1:**
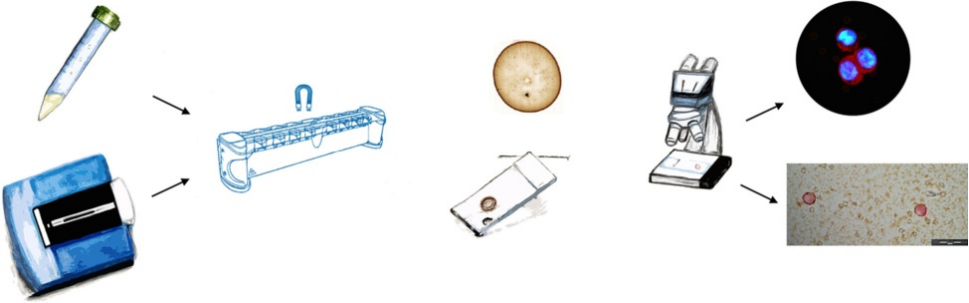
Overview of the method. Enriched CTCs were collected after CellSearch analysis using the Profile or Epithelial cell kit. The solution containing CTCs and leukocytes was placed in a magnetic tray. Following incubation, the non-adherent solvent was removed and the ferrofluid-attached cells were re-suspended in a smaller volume of PBS, thus permitting further enrichment. This solution was dropped and fixed on a glass slide before subsequent staining according to protocols 1 or 2. Visualization is possible with a fluorescence or bright-field microscope, depending on the staining method applied. (Drawing of magnetic stand reprinted with permission from IFI CLAIMS Patent Services.)

The CTC-DropMount method was also used for enriched cells after standard CellSearch epithelial cell analysis (i.e. all cells were semi-automatically stained with CK-phycoerythrin (PE), CD45-allophycocyanin (APC) and DAPI in a procedure described previously (3)). In this case, the solution containing enriched CTCs was extracted from the CellSearch cartridge after complete analyses, and the cartridge was carefully rinsed with 1 × PBS buffer to ensure maximum extraction before transfer to a 1.5 mL Eppendorf tube in a magnetic tray. An overview of the CTC-DropMount method is provided in Figure 1.

### Immunostaining protocol for ERα and HER2

Following cell permeabilization using Dako Target Retrieval solution containing Tris/EDTA buffer solution pH 9.0 and detergent (S2368, Dako Denmark A/S, Glostrup, Denmark), slides were stained according to the optimized protocols detailed in Tables 1 and 2, respectively. Briefly, to define CTCs against the backdrop of remaining leukocytes, a CD45-specific AlexaFluor647-labeled mouse monoclonal antibody (F10894, AbD Serotec, Oxford, UK) was assessed at dilutions of 1:1, 1:5 and 1:10 [32]. The primary antibody used against ERα was a rabbit monoclonal antibody (RM-9101-S1, Thermo Scientific, Fremont, CA, USA), diluted 1:50 [33]. Secondary detection was accomplished with AlexaFluor488-labeled goat anti-rabbit antibody (A-11034, Life Technologies, Carlsbad, CA, USA) at dilutions of 1:200, 1:300, 1:400, and 1:500. For detection of cytokeratin, slides were incubated with CellSearch Staining Reagent containing a PE-labeled mouse monoclonal antibody specific to CK 8, 18 and 19, at a concentration of 0.0006% (Janssen Diagnostics). Antibody dilutions were made with DAKO Antibody Diluent containing 1% FBS in PBS and 0.1% detergent (S2022, Dako). Slides were finally mounted with coverslips and counterstained with an antifade reagent containing the nucleic acid dye, DAPI, (S36942, Life Technologies), thus enhancing resistance to photobleaching.

**Table 1:** **Optimized staining protocol for ERα, CK, and CD45 expression in CTCs and leukocytes**

**Step**	**Reagent**	**Concentration**	**Interval**	**Manufacturer/Batch**
**1. Cell fixation**	Methanol	1:1	5 min at RT	Merck KGaA, Germany, #I659409
**2. Cell permeabilization**	Dako Envision Target Retrieval solution™ (50x) (Tris/EDTA buffer solution, pH 9.0, and detergent)	1:50	20 min at 37°C	Dako Denmark, A/S, #20000821
**3. AB serum**	Dako AB diluent™ (1% FBS in PBS, 0.1% detergent)	1:1	20 min at RT	Dako Denmark, A/S, #00091216
**4** ^**1**^ **. ERα labelling**	Rabbit monoclonal AB specific to ERα	1:50	60 min at 37°C	Thermo Scientific, United States, #9101S1210D
**5** ^**1**^ **. CD45 staining**	Alexa Fluor 647 labeled mouse monoclonal AB specific to CD45	1:5	60 min at 37°C	AbD Serotec, UK, #B173123
**6** ^**2**^ **. ERα staining**	Alexa Fluor 488 labeled goat anti-rabbit secondary AB	1:200	45 min at RT	Life Technologies, United States, #1423009
**7** ^**2**^ **. Cytokeratin 8, 18, and 19 staining**	Phycoerythrin labeled mouse monoclonal AB, specific to CK 8, 18, and 19	0.0006%	45 min at RT	Janssen Diagnostics, United States, #E491A
**8. Nuclear counterstaining**	Slowfade® Gold antifade with nuclear dye, DAPI			Life Technologies, United States, #1500156

Washing with iced PBS 10.0%, v/v, 3 × 3 min between each step.

*Abbreviations:* AB - antibody, DAPI - 4′,6-diamidino-2-phenylindole dihydrochloride, EDTA - Ethylenediaminetetraacetic acid, ERα - estrogen receptorα, PBS - phosphate buffered saline, RT - room temperature, Tris - 2-Amino-2-hydroxymethyl-propane-1,3-diol.

^1^ Step 4 and 5 can be performed simultaneously.

^2^ Step 6 and 7 can be performed simultaneously.

**Table 2:** **Optimized staining protocol for HER2 expression**

**Step**	**Reagent**	**Concentration**	**Interval**	**Manufacturer/Batch**
**1. Cell fixation**	Methanol	1:1	5 min at RT	Merck KGaA, Germany, #I659409
**2. Cell permeabilization**	Dako Envision Target Retrieval solution™ (50×) (Tris/EDTA buffer solution, pH 9.0, and detergent)	1:50	20 min at 37°C	Dako Denmark, A/S, #20000821
**3. AB serum**	Dako AB diluent™ (1% FBS in PBS, 0.1% detergent)	1:1	20 min at RT	Dako Denmark, A/S, #00091216
**4. HER2 labelling**	Rabbit monoclonal AB specific to HER2	1:250	20 min at RT	Abcam plc, United Kingdom, #GR122507-5
**5. ALP conjugation**	ALP-conjugated porcine polyclonal anti-rabbit AB	1:50	30 min at RT	Dako Denmark, A/S, #20008362
**6. LPR**	Red chromogen and substrate buffert	1:100 (chromogen:substrate)	10 min at RT	Dako Denmark, A/S, #10082175
**7. Nuclear counterstaining**	Slowfade® Gold antifade with nuclear dye, DAPI			Life Technologies, United States, #1500156

Washing with iced PBS 10.0% (v/v), 3 × 3 min between each step except after LPR when the slides are only quickly rinsed in with PBS before nuclear counterstaining.

*Abbreviations:* AB, antibody; ALP, Alkaline phosphatase; DAPI, 4′,6-diamidino-2-phenylindole dihydrochloride; EDTA, Ethylenediaminetetraacetic acid; LPR, liquid permanent red; PBS, phosphate buffered saline; RT, room temperature; Tris, 2-Amino-2-hydroxymethyl-propane-1,3-diol.

The level of HER2-expression was investigated using a primary monoclonal rabbit antibody specific for the human HER2 oncoprotein (1:250; EP1045Y, Abcam plc, Cambridge, UK), along with a polyclonal porcine anti-rabbit secondary antibody conjugated with alkaline phosphatase (ALP) (1:50; D0306, Dako). Detection was accomplished based on the ALP-Fast Red reaction, using the substrate and chromogen in Liquid Permanent Red (LPR) (K0640, Dako [34]). Incubation times assessed for the ALP-Fast Red reaction were 5, 10 and, 15 min. LPR was evaluated by both fluorescence and bright-field microscopy.

Immunofluorescence and bright-field analyses were performed with an Olympus BX63 microscope equipped with a dual color/monochrome digital DP80 camera (Olympus Optical CO., Hamburg, Germany). Single pass filters for DAPI, GFP/Alexa488, PE/TxRed and APC/Alexa647/Cy5 were used for staining evaluation. In a few cases, an Olympus BX51 microscope (Olympus Optical CO) and a Zeiss Axio Observer Z1 microscope (Carl Zeiss Microscopy, LLC., NY, USA) outfitted with single pass filters for each individual fluorochrome were used for examination.

### *In vivo* validation

Patient blood samples were investigated for clinical validation of the technique. All patients had metastatic breast cancer and had been included in the ongoing CTC-MBC trial (Clinical Trial Id. NCT01322893) at Lund University, Sweden. In total, nine clinical samples of 7.5 mL whole blood from nine individual patients were assessed by CellSearch profile analysis. CTC-DropMount and subsequent staining according to the staining protocols detailed in Tables 1 and 2 were performed prior to evaluation by fluorescence and bright-field microscopy. All patient sample analyses were processed in conjunction with positive and negative controls, decreasing the risk of methodological errors, as well as confirming successful staining reactions. Ethical permission for the CTC-MBC study was obtained from Lund University Ethical Board (EPN 2010/135) and all patients gave written informed consent.

## Results

### CTC-DropMount

An overview of the CTC-DropMount technique is shown in Figure 1. Using CellSearch Profile analysis from whole blood, the recovery rate was found to be 87% on average (80% for 5 cells, 97% for 15 cells and 84% for 50 cells).

### Immunofluorescence

**Figure 2:**
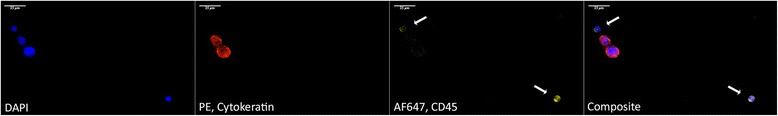
CD45 staining. Secondary staining of cell line cells spiked into healthy donor blood, from left to right: DAPI counterstain (fluorescent blue), cytokeratins 8, 18, and 19 (CK) stained with Phycoerythrin (red), CD45 stained with AlexaFluor 647 (yellow), and a composite of all channels. The two juxtaposed CTCs (CK-positive) stained negative for CD45, while the leukocytes (white arrows) simultaneously stained positive for CD45 and negative for CK, illustrating methodological selectivity.

Using the AlexaFluor647-labeled monoclonal CD45-antibody, it was possible to separate leukocytes from CTCs under standard fluorescence microscopy. This result provides an important prerequisite for further staining and demonstrated sufficient selectivity of the method (see Figure 2). Optimal distinction between CTCs and leukocytes was achieved when combining the two filters for CK-PE and CD45-AlexaFluor647.

**Figure 3:**
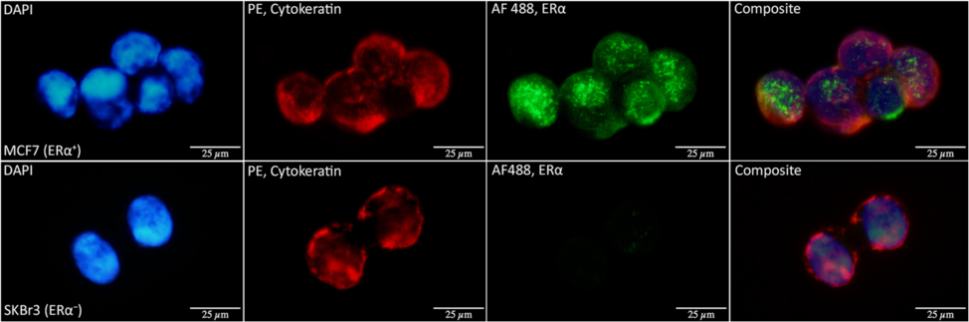
ERα staining of MCF7 and SKBr3 cells. Selective ERα staining demonstrated in MCF7 (ERα^+^) and SKBr3 (ERα^−^) cells. From left to right: DAPI counterstain (fluorescent blue), cytokeratins 8, 18, and 19 (CK) stained with Phycoerythrin (red), estrogen receptor (ERα) stained with AlexaFluor 488 (green), and a composite of all channels. MCF7 showed positive nuclear staining in AlexaFluor 488 indicating positive ERα expression, while SKBr3 was negative.

Criteria for ERα-positivity defined staining of the nuclear region. The process was considered satisfactory when cells in the ERα^+^ cell line (MCF7) consistently stained positive for ERα with low background and marked nuclear intensity, while slides with ERα^−^ cells (SKBr3) simultaneously stained negative. Representative images are displayed in Figure 3.

**Figure 4:**
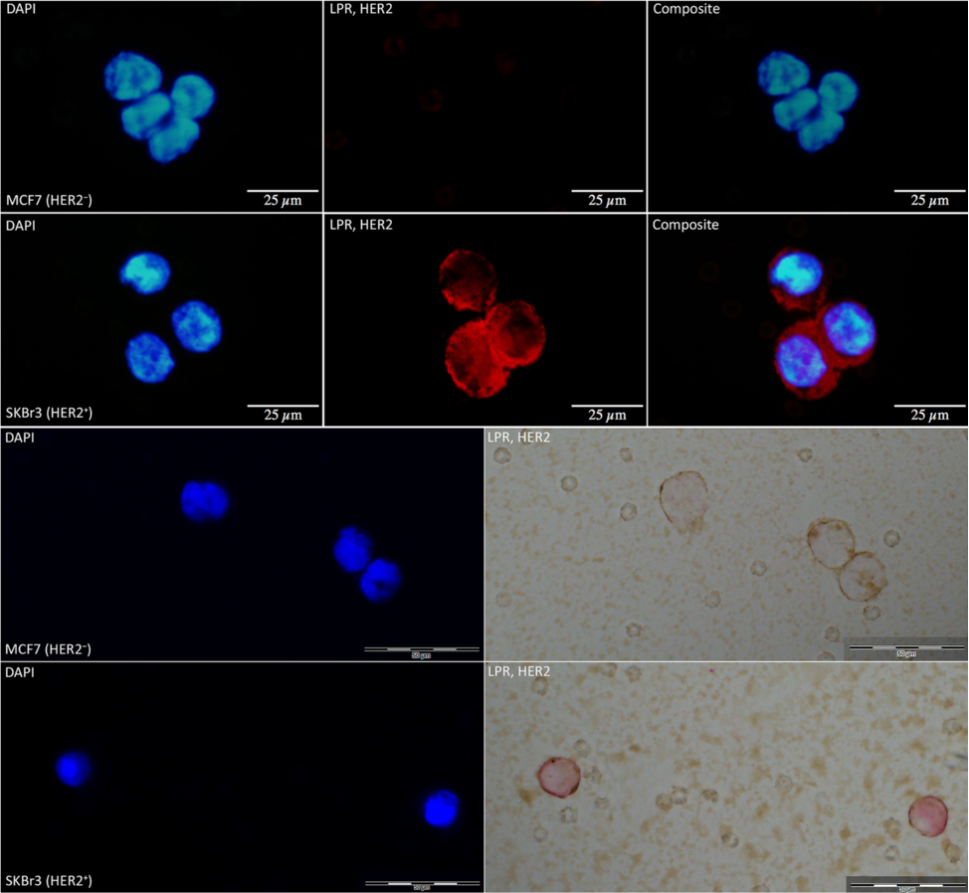
HER2-staining of MCF7 and SKBr3. Selective HER2 staining demonstrated in MCF7 (HER2^−^) and SKBr3 (HER2^+^) cells. First row: MCF7, from left to right: DAPI counterstain (fluorescent blue), HER2 stained with Liquid Permanent Red (red), and a composite of all channels. Second row: SKBr3, in the corresponding channels. Positive membrane staining was visible in SKBr3 cells only. Additionally, assessment of HER2 staining was also possible using bright-field microscopy, as demonstrated in the lower two rows (third row: MCF7, and fourth row: SKBr3).

HER2-staining with LPR proved highly selective (see Figure 4). The ALP-based reaction suits CTCs particularly well since endogenous enzymatic activity is negligible in these samples. Hence, the risk of false positives is insignificant. LPR permits assessment by both fluorescence and bright-field microscopy. Bright-field microscopy has the advantage of being easily accessible in most laboratories and the staining is impervious to fading. Despite the high background caused by ferrofluid remnants from CellSearch analysis, LPR staining was clearly visible. Also, if combined with CK-PE staining, bright-field microscopy was preferable due to the risk of bleed-through between PE and LPR staining in immunofluorescence analyses.

Staining of the fixed cells was optimized mainly using cells from CellSearch Profile analyses where no previous staining and permeabilization had affected the cells. However, the staining procedure was also tested following CellSearch epithelial cell analysis, and although these cells had been previously stained, the results were consistent with previously unstained cells.

**Figure 5:**
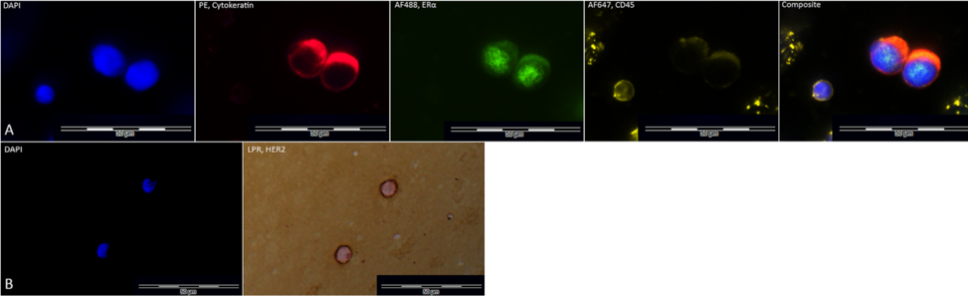
Immunostaining of metastatic breast cancer patient blood samples. Representative images of positive ERα and HER2 staining in clinical samples. Row **A**, from left to right: DAPI counterstain (fluorescent blue), cytokeratins 8, 18, and 19 (CK) stained with Phycoerythrin (red), estrogen receptor (ERα) stained with AlexaFluor 488 (green), and a composite of all channels. This patient sample (no. 4, see Table 3) was collected prior to initiation of therapy, illustrating two clustered ERα^+^ CTCs, adjacent to a solitary leukocyte located in the lower left corner. This patient was diagnosed with an ERα^+^ metastasis. Row **B**, from left to right: DAPI counterstain (fluorescent blue), HER2 stained with Liquid Permanent Red (red). This patient sample (no. 1, see Table 3) was obtained following 6 months of chemotherapy, illustrating HER2^+^ CTCs identified by combination of fluorescence and bright-field microscopy. This patient was diagnosed with a HER2^−^ primary tumor and HER2^−^ metastasis.

The CTC-DropMount method could be confirmed in nine patient samples after CellSearch Profile analysis, and examples of positive ERα and HER2 staining can be found in Figure 5. Table 3 outlines the patients’ characteristics with respect to their primary tumors, metastases, and CTC phenotypes, as well as the total number of CTCs detected by CellSearch. The majority of detected CTCs were negative for both ERα and HER2 expression. We observed considerable intrapatient heterogeneity in levels of biomarker expression and cell morphology, observations that are in concordance with previous research [33]. A visual comparison to corresponding CTCs in the picture galleries from CellSearch epithelial cell analyses suggested that the most intensely stained CTCs were also the most distinctly stained with the CTC-DropMount method for all investigated markers.

**Table 3:** **Patient data for** ***in vivo*** **validation procedures**

**Patient no.**	**Primary tumor phenotype**	**Metastasis phenotype**	Number of CTCs*	**CTC phenotype** ^**§**^
**1**	ER^+^/HER2^**−**^	ER^**−**^/HER2^**−**^	48	ER^**−**^/HER2^+^
**2**	ER^+^/HER2^**−**^	n/a	35	ER^**−**^/HER2^**−**^
**3**	ER^+^/HER2^**−**^	ER^+^/HER2^**−**^	3	n/a^**‡**^
**4**	n/a	ER^+^/HER2^**−**^	12	ER^+^/HER2^**−**^
**5**	ER^**−**^/HER2^+^	n/a	111	ER^**−**^/HER2^**−**^
**6**	ER^**−**^/HER2^+^	n/a	311	ER^**−**^/HER2^**−**^
**7**	ER^+^/HER2^**−**^	ER^+^/HER2^**−**^	107	ER^+^/HER2^**−**^
**8**	ER^+^/HER2^**−**^	ER^+^/HER2^**−**^	0	*Negative control*
**9**	ER^+^/HER2^**−**^	ER^+^/HER2^**−**^	0	*Negative control*

*As defined by CellSearch, single samples assessed 0–6 months from initiation of therapy against metastatic disease.

^§^Phenotype according to CTC-DropMount. Criteria for biomarker positivity were ≥1 ER^+^ CTC, and ≥1 HER2^+^ CTC.

^‡^In this patient no CTCs were identified following secondary staining.

Patients 8, and 9 were selected as negative controls. Neither of these patients had detectable CTCs following secondary staining.

## Discussion

In this study, we present a method for secondary characterization of breast cancer CTCs after CellSearch analysis. Protocols for the clinically important predictive markers ERα and HER2 were optimized in breast cancer cell lines and subsequently verified in samples from patients with metastatic breast cancer. Fixation of CTCs was performed with the CTC-DropMount method described here, and ERα and HER2 staining protocols proved selective and consistent in our *in vitro* model system.

Secondary phenotypic characterization of fixed CTCs on standard microscope slides provides the possibility of concurrent morphological evaluation, assessment of the total number of cells and an estimation of the fraction of CTCs with expression of the analyzed biomarker. This gives unique information on the heterogeneity of marker expression, which is not available using PCR-based molecular methods, for example [28,35,36]. Assessment of ERα status in CTCs could identify patients eligible for endocrine treatment that otherwise may be overlooked (i.e. ERα^−^ primary tumor/ERα^+^ CTCs). Two of the nine patients included in the *in vivo* validation experiments presented ERα^+^ CTCs (see Table 3, and representative images in Figure 5). Both of these samples were drawn at or just prior to initiation of treatment against metastatic disease. The phenotype of the primary tumor from one patient was classified as ERα^+^, while the second patient had a confirmed ERα^+^ metastatic biopsy (Table 3). Conversely, detection of ERα^−^ CTCs in a patient with an ERα^+^ primary tumor might, in part, explain the lack of treatment response observed in this cohort. A similar assumption regarding HER2 gene amplification seems reasonable, since a subset of patients acquire oncogene amplification during disease progression [30]. The true number of patients suited for HER2-targeted treatment may in fact be higher than the number treated at present. This is currently being investigated in the ongoing European DETECT III and CIRCE T-DM1 studies, where the CTC HER2-positive phenotype is used as a treatment predictive marker [31]. In this study, HER2^+^ CTCs were identified after 6 months of chemotherapy in a metastatic breast cancer patient with a HER2^−^ primary tumor and a HER2^−^ metastasis biopsy (see Table 3 and Figure 5).

A few previous studies have used immunological staining methods for secondary phenotypic characterization. Swennenhuis *et al.* fixed CTCs within the cartridge after complete CellSearch analysis using immunofluorescence and FISH analysis for successful characterization of HER2-status [37]. However, the CellTracks II analyzer had to be modified to improve the resolution and light collection. Other studies have used the FITC-channel in the CellSearch system, where the intensity of HER2 staining is scored as negative (0), very weak (1+), moderate (2+) or very bright (3+) [27,38,39]. The clinical value of specific cut-off thresholds remains to be determined. Paoletti *et al.* recently reported a method utilizing the CellSearch-integrated FITC-channel for analysis of ERα, HER2, Ki67, and BCL-2 in individual blood samples with the intention of predicting resistance to endocrine therapy [40]. By implementing this approach, 7.5 mL of blood is required for analysis of each respective biomarker. Few studies have described methods for secondary characterization of ERα and HER2 in CTCs after Ficoll density gradient separation and cytospin preparations [17,33]. An advantage in circumventing immunological enrichment before fixation of the cells onto microscope slides is the exemption from EpCAM-dependent selection. On the other hand, the number of cells that have to be screened manually by standard microscopy is very high, thus hampering the clinical feasibility and cost effectiveness if introduced into routine clinical practice.

Fixation of CTCs on microscope slides with the described CTC-DropMount method provides the possibility to use a standard fluorescence microscope for CTC characterization after immunological enrichment with the FDA-cleared CellSearch system. An advantage of the described method for secondary characterization is the scope to expand the CTC analysis to other putative predictive markers as well as to more experimental markers, for example stem cell markers, epithelial mesenchymal transition (EMT) markers or markers associated with metastasis, proliferation or apoptosis, thus increasing our knowledge of metastasis biology. Selection of single CTCs or a subset of CTCs is also possible after CTC-DropMount using laser capture microdissection, for example. Subsequent single cell genomic analyses could open the door to an even more detailed molecular characterization of CTCs [41].

The informative advantage of heterogeneity in marker expression using secondary staining methods is also associated with the need for a prognostic cut-off value for the fraction or intensity of expression within the CTC population [17]. Using different cut-offs for marker positivity has given conflicting results regarding discordance of marker expression between primary tumors and CTCs [17,33,42]. Thus, the prognostic significance of marker heterogeneity in breast cancer CTCs has to be determined, and reliability on the staining methodology is of immense importance. Using the CTC-DropMount technique, we found distinct nuclear staining of ERα in the MCF7 cell line and used nuclear staining as a criterion for ERα-positivity. However, to effectively determine the clinical implications, defined criteria, such as the number of CTCs to be evaluated and the fraction of ERα- or HER2-positive CTCs, has to be decided in future clinical studies.

The cell recovery rate after CTC-DropMount fixation was 87%, which is at the high end of recovery compared with studies using different methods for enrichment, fixation and detection [33,43-48]. The 80% recovery rate of five spiked cell line cells further indicates that this method could be useful in the clinical setting, where number of CTCs at the established CellSearch cut-off value (≥5 CTCs in metastatic breast cancer) is common.

## Conclusions

In conclusion, our results indicate that by retrieval of a single blood sample from patients with metastatic breast cancer it is possible to ascertain the status of important predictive biomarkers expressed in breast cancer CTCs. The discordance of expression between primary tumors and metastases urgently informs us that new diagnostic tools are required for optimal treatment selection in both primary and metastatic breast cancer.

## Abbreviations

ALP: Alkaline phosphatase
APC: Allophycocyanin
CK: Cytokeratins
CTCs: Circulating tumor cells
DAPI: 4′,6-diamidino-2-phenylindole
EDTA: Ethylenediaminetetraacetic acid
EpCAM: Epithelial cell adhesion molecule
ERα: Estrogen receptor α
FBS: Fetal bovine serum
HER2: Human epidermal growth factor receptor 2
LPR: Liquid permanent red
PBS: Phosphate buffered saline
PE: Phycoerythrin
Tris: 2-Amino-2-hydroxymethylpropane-1,3-diol
